# Effect of maximum exercise on left ventricular deformation and its correlation with cardiopulmonary exercise capacity in competitive athletes

**DOI:** 10.1186/s44156-023-00029-6

**Published:** 2023-10-04

**Authors:** J. Kandels, S. Stöbe, A. Kogel, P. Hepp, H. Riepenhof, J. N. Droste, T. Stoeggl, R. P. Marshall, U. Rudolph, U. Laufs, S. Fikenzer, A. Hagendorff

**Affiliations:** 1 Klinik und Poliklinik für Kardiologie, Universitätsklinikum Leipzig, Liebigstr. 20, 04103 Leipzig, Germany; 2 Klinik und Poliklinik für Orthopädie, Unfallchirurgie Und Plastische Chirurgie, Universitätsklinikum, 04103 Leipzig, Germany; 3 RasenBallsport Leipzig GmbH, Cottaweg 3, 04177 Leipzig, Germany; 4 Center for Rehabilitation and Sports Medicine, BG Klinikum Hamburg, 21033 Hamburg, Germany; 5 Red Bull Athlete Performance Center, 5303 Salzburg, Austria; 6 Department of Sport and Exercise Science, Universität Salzburg, 5020 Salzburg, Austria; 7 Department of Orthopedic and Trauma Surgery, Martin-Luther-University Halle-Wittenberg, 06120 Halle, Germany

**Keywords:** Echocardiography, Athletes, Cardiopulmonary exercise test, Deformation, Longitudinal strain, Work index

## Abstract

**Background:**

Global longitudinal strain (GLS) and global myocardial work index (GWI) allow early detection of subclinical changes in left ventricular (LV) systolic function. The aim of the study was to investigate the immediate effects of maximum physical exercise by different exercise testing methods on early post exercise LV deformation parameters in competitive athletes and to analyze their correlation with cardiopulmonary exercise capacity.

**Methods:**

To reach maximum physical exercise, cardiopulmonary exercise testing (CPET) was performed by semi-recumbent ergometer in competitive handball players (n = 13) and by treadmill testing in competitive football players (n = 19). Maximum oxygen uptake (VO_2max_) indexed to body weight (relative VO_2max_) was measured in all athletes. Transthoracic echocardiography and blood pressure measurements were performed at rest and 5 min after CPET in all athletes. GLS, GWI and their changes before and after CPET (ΔGLS, ΔGWI) were correlated with (relative) VO_2max_.

**Results:**

In handball and football players, GLS and GWI did not differ significantly before and after CPET. There were no significant correlations between GLS and relative VO_2max_, but moderate correlations were found between ΔGWI and relative VO_2max_ in handball (r = 0.631; *P* = 0.021) and football players (r = 0.592; *P* = 0.008). Furthermore, handball (46.7 ml/min*kg ± 4.7 ml/min*kg vs. 37.4 ml/min*kg ± 4.2; *P* = 0.004) and football players (58.3 ml/min*kg ± 3.7 ml/min*kg vs. 49.7 ml/min*kg ± 6.8; *P* = 0.002) with an increased ΔGWI after CPET showed a significant higher relative VO_2max_.

**Conclusion:**

Maximum physical exercise has an immediate effect on LV deformation, irrespective of the used testing method. The correlation of relative VO_2max_ with ΔGWI in the early post exercise period, identifies ΔGWI as an echocardiographic parameter for characterizing the current individual training status of athletes.

## Introduction

In competitive athletes, transthoracic echocardiography (TTE) is a widely used noninvasive imaging modality that allows the assessment of left ventricular (LV) systolic function by LV ejection fraction (EF) and by LV deformation analysis by speckle tracking [1, 2].

Global longitudinal strain (GLS) is defined as the percentage longitudinal shortening (change in length compared to baseline length) during systole. GLS allows early detection of subclinical myocardial damage, e.g., due to myocardial fibrosis [3–8]. Normal GLS values range from − 16.0 to − 22.0%. Unfortunately GLS is significantly affected by afterload conditions [9].

A relatively new echocardiographic approach involves the assessment of non-invasive pressure strain loops (PSL). The global myocardial work index (GWI) is a modern echocardiographic parameter which is based on PSL and combines myocardial deformation imaging using 2D speckle tracking with non-invasive afterload determination using brachial cuff blood pressure measurement [10]. Values of GWI will automatically be calculated by the respective program after speckle tracking analyses have been performed and blood pressure values have been entered. Russell et al. repeatedly demonstrated that loop areas by invasively measured LV pressure and speckle-tracking echocardiography were identical compared with the non-invasive method by estimated LV pressure and speckle-tracking echocardiography [10]. GWI has proven to be afterload-independent and related to myocardial deformation and contractile function [11, 12]. For this reason, GWI might offer advantages over GLS in athletes exposed to different afterload conditions during exercise. Normal values of GWI range from 1900 to 2100 mmHg% [9]. In contrast to LVEF, GLS and GWI have been shown to be reliable in distinguishing between physiological adaption (e.g., LV hypertrophy due to exercise) and pathological changes (e.g., hypertrophic cardiomyopathy) in the athletes’ heart [13, 14].

Cardiopulmonary exercise testing (CPET) is an established method to characterize the pulmonary, vascular, musculoskeletal, and cardiac system in competitive athletes. Maximum oxygen uptake (VO_2max_) is considered the international standard for determining physical capacity [15, 16]. CPET can be helpful to assess and optimize the athlete’s current training condition [17–19]. Since VO_2max_ is highly dependent on the athlete’s body type, it is often indexed to body weight (relative VO_2max_). In addition to the athlete’s body type, age, gender, and sport type also appear to have a significant impact on VO_2max._ In healthy males aged 18 and 30 years, a mean relative VO_2max_ of 48 ml/min*kg has been reported [20].

In general CPET is performed using a cycle ergometer or treadmill [21, 22]. The cycle ergometer is well applicable in patients with unfavorable conditions (e.g., obesity, joint issues, deconditioning) and allows convenient intra-test procedures (ECG, blood pressure, blood sampling) due to less movement artefacts. Treadmill ergometry is more susceptible to movement artefacts, yet it allows running at predefined speed and incline, activates more muscle groups and can lead to higher levels of peak oxygen uptake [22, 23].

The objective of the present study was to investigate the effect of maximum physical exercise by different testing methods on LV deformation in competitive athletes and to analyze the relationship between cardiopulmonary exercise capacity and LV deformation parameters. We hypothesized that the effect of maximum physical exercise on LV deformation does not differ between the two testing methods and GWI can be used as a surrogate parameter for VO_2max_.

## Methods

### Study population and study design

The study population (n = 32) was composed of competitive handball (n = 13) and football (n = 19) players from the first handball and football division in Germany. Handball and football players were considered separately because of differences in body size and constitution. Furthermore, the test modalities at the corresponding centers were different, so that this division seemed reasonable. Due to their different physical constitution and the cardiovascular demands resulting from different types of exercise. All athletes provided informed consent after full explanation of the purpose and order of all procedures. The study was conducted in accordance with the Declaration of Helsinki and was approved by the ethical committee of the University of Leipzig (073/18-ek).

All athletes were enrolled in the outpatient clinic of cardiology from May until July 2020 (handball players) and in July 2021 (football players). They were tested for SARS-CoV-2 and had a negative PCR test taken at most 48 h before the examination. All athletes were asymptomatic and completely free of cardiovascular diseases or risk factors. A physical examination including vital parameters was performed in all subjects. Further, an electrocardiogram at rest, incremental CPET, and TTE (before and 5 min after CPET) were performed. Blood pressure was assessed by non-invasive brachial cuff measurement in supine position at rest and 5 min after CPET simultaneously with the acquisition of apical views by TTE. The examination protocol of the athletes (semi-recumbent vs. treadmill testing) was set by the respective sports federations. Logistic procedures of CPET and TTE examinations were limited by the surrounding conditions at the corresponding centers. The workflow after completion of CPET and before the beginning of post-exercise TTE was supervised by two non-medical assistants. TTEs were performed by two physicians with two ultrasound machines, so that the post-exercise TTE could be performed as soon as possible in all athletes, within a maximum of 5 min. Some restructuring was necessary with the aim of performing both TTE (at rest and after CPET) in a standardized manner under the same conditions in left lateral position to avoid methodological effects on LV deformation, e.g., effects of changes in position (upright posture vs. left lateral position). Image acquisition of post-exercise TTE was focused on strain measurements, so that cineloops of apical, midventricular, and basal parasternal short-axis views, a LVOT Doppler spectrum for timing of aortic valve opening (AVO) and closure (AVC) all three standardized apical views were acquired within a maximum of 3 min. Each sequence was documented 3 times. Thus, the acquisition time of these 19 cineloops averaged 2 min.

### Incremental cardiopulmonary exercise test for handball players

CPET was performed on a semi-recumbent ergometer (GE eBike, GE Healthcare GmbH, Solingen, Germany) at a constant speed of 60–70 revolutions per minute (rpm). The semi-supine angle was about 45° without lateral rotation in all handball players. The test started at a workload of 50W with an increase of 50W every 3 min until volitional exhaustion occurred. Each subject continued for an additional 5-min recovery period at a workload of 25W. In the CPET, ergospirometry data were collected using a digital spirometer (Vyntus™ CPX, Vyaire Germany, Hoechberg, Germany). Absolut and relative oxygen consumption (VO_2max_) were assessed to characterize the respiratory function of athletes. VO_2max_, minute ventilation (VE), and heart rate (HR) (GE Cardiosoft, GE Healthcare GmbH, Solingen, Germany) were monitored continuously at rest, during CPET, and during recovery. In addition to VO_2max_ the individual fitness index for weight-independent comparisons of athletes’ cardiopulmonary exercise capacity was calculated by the following: absolute VO_2max_/(weight^0,73^) [24].

### Incremental cardiopulmonary exercise test for football players

Run performance diagnostics were performed on a treadmill (HP Cosmos, Traunstein, Germany) to determine VO_2max_ (Comsed, Rome, Italy), peak performance (P_peak_), maximum heart rate (HR_max_), and lactate threshold (LT), as well as running economy and fractional utilization of VO_2max_ at LT. The testing protocol contained a 2-phase test consisting of an incremental, sub-maximal exercise test (phase 1) followed by a ramp test (phase 2) interspersed with an 8 min break (4 min active walking at 4 km/h followed by 4 min passive rest) [25].

Athletes started the incremental test at 8.0 km/h at a treadmill incline of 0%. The test was completed when (i) blood lactate has increased by ≥ 1 mmol/L compared to the previous stage, (ii) Borg value was > 17 (on the 6–20 scale), (iii) the respiratory exchange ratio was > 1.0 in two consecutive stages. Criteria (ii) and (iii) were introduced to prevent athletes who do not achieve a blood lactate increase of ≥ 1 mmol/L from becoming prematurely exhausted before the upcoming ramp protocol. The last stage was terminated when the result of the blood lactate level of the previous stage was displayed and was ≥ 1 mmol/L compared with the penultimate stage (generally 1 to 1.5 min). The start speed of the ramp test and the speed of LT determined in the incremental test (equal to the speed of the stage before the lactate increase of ≥ 1 mmol/L) were increased by 1 km/h every minute until voluntary exhaustion.

### Transthoracic echocardiography

TTE was performed using a Vivid E9 or E95 ultrasound system with a 4Vc phased array probe (GE Healthcare Vingmed Ultrasound AS, Horten, Norway). Post-processing analyses were performed with the EchoPac software (Version 203, GE Healthcare Vingmed Ultrasound AS, Horten, Norway). LV morphology was characterized by LV dimensions (M-Mode) including LV length, relative wall thickness (RWT), LV mass (LVM) (by the Devereux formula), and LV mass index (LVMi) according to current recommendations [26]. LV systolic function was characterized by LVEF based on LV end-diastolic (LVEDV) and end-systolic volume (LVESV) assessed by LV biplane planimetry by the modified Simpson’s rule in the apical 2- and 4-chamber view as well as by Cardiac Index (CI) (by Doppler echocardiography) [27]. Myocardial deformation was characterized by GLS using 2D speckle tracking analysis of the apical long axis-, 2-, and 4-chamber-view according to current recommendations [3, 4, 28]. The endocardial contour was manually adjusted, whereas only segments with accurate tracking were accepted. Tracking areas were manually adjusted to enable full myocardial tracking.

Additionally, GWI was calculated by using the longitudinal strain analysis of the apical LV long axis-, 2-, and 4-chamber-view coupled with the noninvasive blood pressure measurements to attain a pressure-strain loop of the LV [10]. This analysis was performed by post-processing using EchoPac software. In all athletes, the change in GLS (ΔGLS) and GWI (ΔGWI) was calculated by the difference between pre and post CPET values.

Diastolic function was characterized by maximum blood flow velocities (V_max_) of E- and A-wave, E/A-ratio, myocardial V_max_ of e′ and a′ of the basal septal and lateral mitral annulus, septal and lateral E/e′-ratio (including average E′/e′-ratio (septal and lateral)) and systolic pulmonary artery pressure (sPAP) according to current recommendations [29]. In all athletes three cardiac cycles were assessed according to current literature [30].

### Statistical analysis

All statistical analyses were performed using SPSS Statistics (version 24.0, IBM, Armonk, NY) and Microsoft Office Excel (version 16.53, Microsoft). Continuous variables were expressed as mean value ± standard deviation (SD). Further, percentage changes after CPET compared to resting conditions were stated. In consideration of the small sample size, we decided to forgo distribution analyses. Statistical significance was accepted for *P* value < 0.05. Student’s t-test was used to compare the echocardiographic results before and after CPET.

Pearson correlation coefficient *r* was used to test the correlation between different echocardiographic parameters at rest, after CPET and for the percentage change of each parameter after CPET compared to resting conditions: *r* ≤ 0.5 (poor correlation), *r* = 0.5–0.7 (moderate correlation) and *r* ≥ 0.7 (good correlation).

Intra- and interobserver variabilities of main echocardiographic parameters (LV volumes, LVEF, GLS, CI, sPAP) were assessed in randomly selected athletes (n = 10). The second investigator used the same datasets, and both were blinded to each other’s results.

## Results

Baseline characteristics of handball and football players are shown in Table 1.

**Table 1:** Baseline characteristics

Mean values ± SD	Handball players (Semi-recumbent ergometer)			Football players (Treadmill)		
	At rest	After CPET	*P* value	At rest	After CPET	*P* value
Age (year)	25.2 ± 3.5	–		22.1 ± 4.7	–	
Sex (% of male)	13 (100)	–		19 (100%)	–	
Weight (kg)	98.4 ± 8.2	–	–	79.1 ± 10.6	–	–
Height (cm)	193.3 ± 7.5	–	–	181.5 ± 7.8	–	–
BSA (m^2^)	2.3 ± 0.1	–	–	1.99 ± 0.17	–	–
BMI (kg/m^2^)	26.3 ± 1.7	–	–	23.9 ± 2.0	–	–
BPs (mmHg)	126.0 ± 10.5	152.6 ± 14.0	< 0.001*	133.8 ± 11.1	149.0 ± 14.7	< 0.001*
BPd (mmHg)	80.8 ± 7.3	65.5 ± 9.7	< 0.001*	82.2 ± 7.7	78.0 ± 10.2	0.090
HR (bpm)	68 ± 9	91 ± 13	< 0.001*	64 ± 12	91 ± 14	< 0.001*

*Statistically significant (p < 0.05). SD: standard deviation; BSA: body surface area; BMI: body mass index; BPs: systolic blood pressure; BPd: diastolic blood pressure; HR: heart rate; CPET: cardiopulmonary exercise testing

### Semi-recumbent ergometer

Left ventricular volumes were significantly lower after physical exertion compared to resting conditions (Table 2). Left ventricular ejection fraction was similar before and after CPET (Table 2). E/A-ratio was significantly decreased after physical exertion, mainly due to a reduction of the A-wave (Table 3). Myocardial early (e′) diastolic tissue velocities were significantly lower, although this did not lead to a reduction of E/e′, mainly due to consistent passive diastolic filling velocities (E-wave) (Table 3). After CPET sPAP was still in normal ranges.

**Table 2:** Conventional echocardiographic parameters of left ventricular morphology and function

Mean values ± SD	Handball players (Semi-recumbent ergometer)			Football players (Treadmill)		
	At rest	After CPET	*P* value	At rest	After CPET	*P* value
IVSD (mm)	9.9 ± 0.9	10.5 ± 1.1	0.073	9.8 ± 1.8	10.8 ± 1.5	0.009*
PWD (mm)	9.7 ± 1.9	10.0 ± 1.0	0.301	9.4 ± 1.9	9.8 ± 2.5	0.494
LVEDD (mm)	57.5 ± 3.8	54.5 ± 3.5	0.001*	57.5 ± 4.2	53.4 ± 4.1	< 0.001*
LVESD (mm)	35.0 ± 3.3	34.1 ± 3.4	0.359	36.4 ± 4.8	34.5 ± 3.9	0.048*
LVL (mm)	92.9 ± 5.7	91.1 ± 7.5	0.404	91.3 ± 5.6	88.0 ± 5.6	0.0194*
LVM (g)	215.1 ± 39.7	215.0 ± 39.5	0.993	219.4 ± 55.9	221.0 ± 43.0	0.873
LVMI (g/m^2^)	62.1 ± 3.6	63.1 ± 7.4	0.349	101.4 ± 22.6	110.8 ± 20.3	0.059
RWT	0.32 ± 0.06	0.37 ± 0.04	< 0.001*	0.32 ± 0.07	0.39 ± 0.07	< 0.001*
LVEDV (ml)	162.9 ± 23.5	145.2 ± 20.9	0.010*	164.8 ± 26.8	138.6 ± 24.1	< 0.001*
LVESV (ml)	51.2 ± 11.5	48.5 ± 11.2	0.402	57.5 ± 17.7	50.0 ± 12.7	0.019*
LVSV (ml)	111.7 ± 16.3	96.7 ± 16.4	0.006*	107.3 ± 15.3	88.6 ± 15.0	< 0.001*
EF (%)	68.6 ± 4.4	66.6 ± 6.0	0.089	65.8 ± 6.9	64.3 ± 5.6	0.258
CI (l/m^2^)	3.1 ± 0.7	3.9 ± 0.8	< 0.001*	3.1 ± 0.6	4.1 ± 0.8	< 0.001*
GLS (%)	− 18.8 ± 1.6	− 18.1 ± 1.7	0.079	− 18.3 ± 1.7	− 17.7 ± 1.6	0.119
GWI (mmHg%)	1837.7 ± 316.0	1974.7 ± 222.6	0.197	1899.3 ± 280.7	1963.5 ± 370.0	0.461

*Statistically significant (p < 0.05). SD: standard deviation; IVSD: Interventricular septum diameter; PWD: Posterior wall diameter; LVEDD: left ventricular end-diastolic diameter; LVESD: left ventricular end-systolic diameter; LVL: left ventricular length; LVM: left ventricular mass; LVMi: LVM index; RWT: relative wall thickness; LVEDV: left ventricular end-diastolic volume; LVESV: left ventricular end-systolic volume; LVSV: left ventricular stroke volume; EF: ejection fraction; CI: cardiac index; GLS: global longitudinal strain; GWI: Global myocardial work index

**Table 3:** Parameters of left ventricular diastolic and right ventricular function

Mean values ± SD	Handball players (Semi-recumbent ergometer)			Football players (Treadmill)		
	At rest	After CPET	*P* value	At rest	After CPET	*P* value
E-wave (m/s)	0.76 ± 0.15	0.72 ± 0.16	0.385	0.73 ± 0.16	0.71 ± 0.20	0.668
A-wave (m/s)	0.43 ± 0.08	0.64 ± 0.19	0.002*	0.41 ± 0.08	0.60 ± 0.19	< 0.001*
E/A-ratio	1.82 ± 0.50	1.19 ± 0.40	< 0.001*	1.87 ± 0.67	1.24 ± 0.35	< 0.001*
Average e′	0.16 ± 0.04	0.14 ± 0.02	0.004*	0.15 ± 0.02	0.13 ± 0.02	< 0.001*
Average a′	0.08 ± 0.01	0.09 ± 0.02	0.097	0.07 ± 0.01	0.09 ± 0.02	< 0.001*
Average e′/a′-ratio	1.90 ± 0.29	1.59 ± 0.37	0.011*	1.78 ± 0.49	1.49 ± 0.50	0.021*
Average E/e′-ratio	4.94 ± 1.11	5.09 ± 1.01	0.602	4.94 ± 1.17	5.39 ± 1.28	0.007*
TAPSE (cm)	2.1 ± 1.3	2.0 ± 0.9	0.652	1.9 ± 0.7	2.0 ± 1.2	0.757
sPAP (mmHg)	25.0 ± 4.3	23.8 ± 3.5	0.879	23.7 ± 2.9	22.5 ± 1.8	0.309

*Statistically significant (p < 0.05). SD: standard deviation

**Fig. 1:**
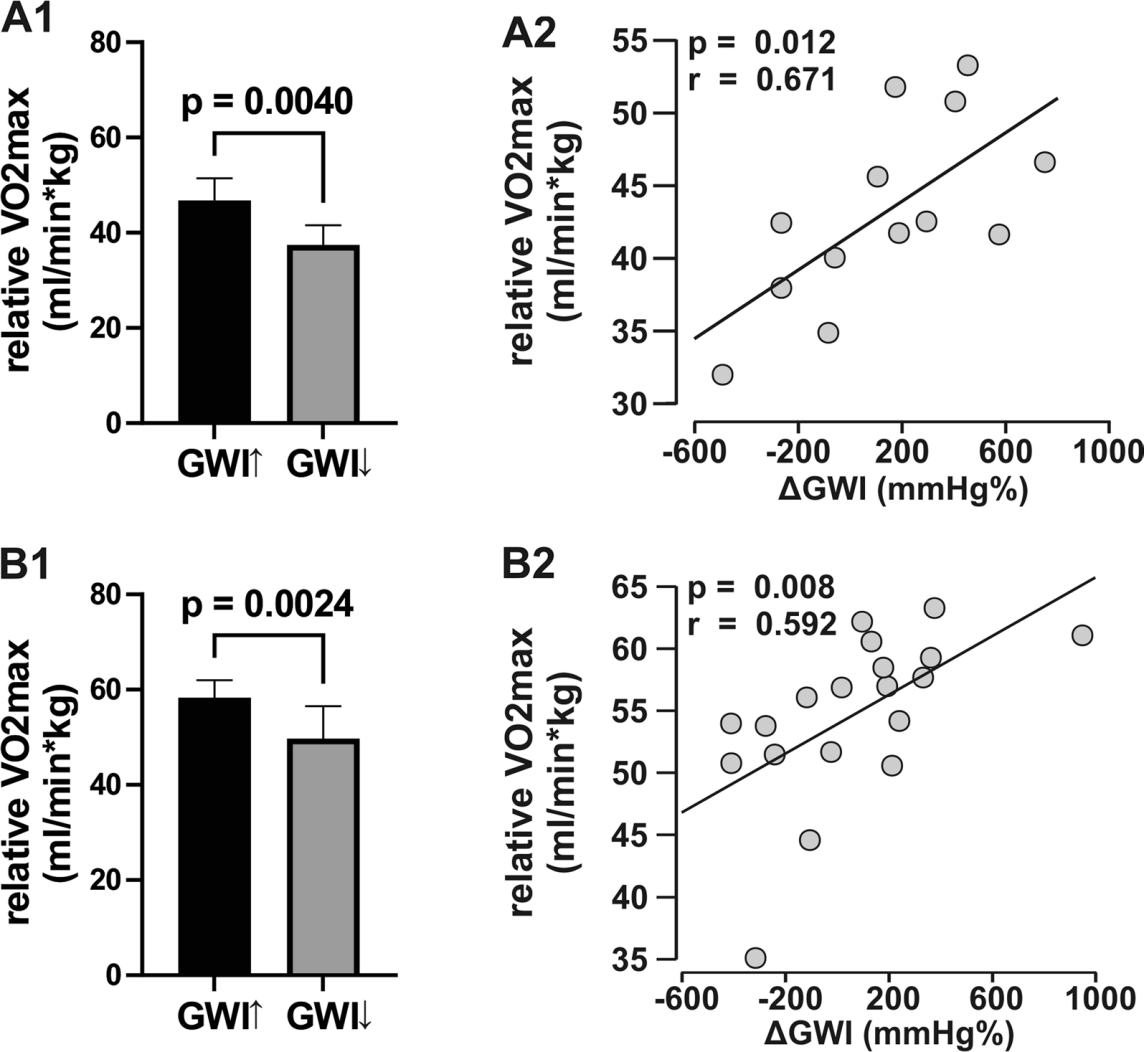
Relative VO_2max_ in competitive handball (**A1**) and football players (**B1**) with increased (↑) global myocardial work index (GWI) after cardiopulmonary exercise test (CPET) compared to the athletes with decreased GWI (↓). The correlation between the change of GWI (ΔGWI) after CPET with relative VO_2max_ as a surrogate parameter for cardiopulmonary exercise capacity in competitive handball (**A2**) and football players (**B2**)

Global longitudinal strain (− 18.8 ± 1.6% vs. − 18.1 ± 1.7%; *P* = 0.079) and GWI did not differ before and after CPET (1838 ± 316 mmHg% vs. 1975 ± 223 mmHg%; *P* = 0.197). Specifically, GWI increased in eight and decreased in five handball players after CPET (Fig. 1). Athletes with an increase in GWI after CPET showed higher relative VO_2max_ values (46.7 ml/min*kg ± 4.7 ml/min*kg vs. 37.4 ml/min*kg ± 4.2; *P* = 0.004) (Fig. 1). Athletes with a decrease in GWI after CPET had the lowest relative VO_2max_ values (Fig. 1). At maximum physical exercise VO_2max_ was 4214 ± 489 ml/min and relative VO_2max_ was 43.1 ± 6.4 ml/min*kg. Calculated fitness index was 149 ± 20 ml/min*kg.

No correlation with VO_2max_ or relative VO_2max_ was shown for GLS at rest, after CPET, and ΔGLS. In contrast, there was a correlation between GWI after CPET and VO_2max_ (r = 0.631; *P* = 0.021), ΔGWI and VO_2max_ (r = 0.762; *P* = 0.002), as well as ΔGWI and relative VO_2max_ (r = 0.671; *P* = 0.012; Table 4).

**Table 4:** Correlations between left ventricular deformation and cardiopulmonary exercise capacity

Mean values ± SD	Handball players (Semi-recumbent ergometer)		Football players (Treadmill)	
	Pearson’s R	P value	Pearson’s R	P value
VO_2max_ vs. GLS at rest	0.047	0.880	0.219	0.367
VO_2max_ vs. GLS after CPET	− 0.067	0.828	− 0.041	0.869
VO_2max_ vs. ΔGLS	− 0.136	0.657	− 0.325	0.175
Relative VO_2max_ vs. GLS at rest	− 0.019	0.952	0.008	0.974
Relative VO_2max_ vs. GLS after CPET	0.112	0.716	0.094	0.701
Relative VO_2max_ vs. ΔGLS	0.159	0.605	0.093	0.706
VO_2max_ vs. GWI at rest	− 0.428	0.144	0.031	0.901
VO_2max_ vs. GWI after CPET	0.631	0.021*	0.336	0.160
VO_2max_ vs. ΔGWI	0.762	0.002*	0.346	0.147
Relative VO_2max_ vs. GWI at rest	− 0.415	0.159	− 0.099	0.688
Relative VO_2max_ vs. GWI after CPET	0.502	0.080	0.459	0.048*
Relative VO_2max_ vs. ΔGWI	0.671	0.012*	0.592	0.008*

^*^Statistically significant (p < 0.05). SD: standard deviation; VO_2max_: maximum oxygen uptake; GLS: global longitudinal strain; GWI: global myocardial work index; CPET: cardiopulmonary exercise testing; Δ: change before and after CPET

### Treadmill testing

Left ventricular volumes were significantly lower after physical exertion compared to resting conditions (Table 2). Left ventricular ejection fraction was similar before and after CPET (Table 2). E/A-ratio and E/e′ were significantly lower after CPET (Table 2). sPAP was in normal ranges before and after CPET.

Global longitudinal strain (− 18.3 ± 1.7% vs. − 17.7 ± 1.6%; *P* = 0.119) and GWI did not differ before and after CPET (1899 ± 281 mmHg% vs. 1963 ± 370 mmHg%; *P* = 0.461). GWI increased in 11 and decreased in eight football players after CPET (Fig. 1). Athletes with an increase in GWI after CPET showed higher relative VO_2max_ values (58.3 ml/min*kg ± 3.7 ml/min*kg vs. 49.7 ml/min*kg ± 6.8; *P* = 0.002) (Fig. 1). At maximum physical exercise VO_2max_ was 4306 ± 594 ml/min and relative VO_2max_ was 54.7 ± 6.5 ml/min*kg. Calculated fitness index was 178 ± 20 ml/min*kg.

No correlation with VO_2max_ or relative VO_2max_ was shown for GLS at rest, after CPET, and ΔGLS. However, GWI after CPET and relative VO_2max_ (r = 0.459; *P* = 0.048) as well as **Δ**GWI and relative VO_2max_ (r = 0.592; *P* = 0.008) showed moderate correlations (Table 4)_._

### Intra- and interobserver variabilities

Intraobserver variabilities of GLS measurements were 2.16% at rest (*P* = 0.638) and 2.61% after CPET (*P* = 0.491). Interobserver variabilities of GLS measurements were 3.91% at rest (*P* = 0.337) and 4.23% after CPET (*P* = 0.312). Intra- and interobserver variabilities for LV volumes, LVEF, CI, and sPAP measurements were < 5% without reaching statistical significance.

## Discussion

The main findings of the present study are: (1) GLS and GWI did not differ significantly before and after semi-recumbent ergometer and treadmill testing. (2) There was no significant correlation between GLS and (relative) VO_2max_, but (3) there were significant correlations between ΔGWI and relative VO_2max_ in semi-recumbent ergometer and treadmill testing.

### Baseline echocardiographic parameters

Changes of conventional echocardiographic parameters after CPET (e.g. LV volumes), were in line with the results of previous studies and have already been described [31]. Both, low intra- and interobserver variabilities highlight the quality of data acquisition as well as the robustness of conventional but also deformation parameters, e.g. GLS [7].

### Impact of maximum exercise on early post exercise global longitudinal strain

The impact of pre- and afterload conditions on LV systolic function has already been described [32]. In general data analyzing the impact of physical stress on LV deformation are scarce and the results of previous studies are highly heterogeneous. Some previous clinical studies have proven a significant impact of pre- and afterload conditions on GLS [33–36]. Nevertheless, GLS was not able to predict load-independent contractility in a porcine model [37]. The impact of different CPET methods on global longitudinal strain in competitive athletes has not been described before.

Liang et al. assessed GLS in 15 swimming athletes before and after high-intensity exercise, where GLS was significantly lower after high-intensity exercise [38]. The decrease of GLS was explained by negative effects on myocardial cells based on anaerobic glycolysis due to ischemia, hypoxia, and the formation of lactic acid with a reduction of myocardial contraction force and consequently, a decrease of LV myocardial contractile function [39]. These results were in contrast to Gruca et al., where GLS was significantly increased in 69% of 111 male elite basketball players in the first minute after maximum physical exertion due to treadmill testing [40]. The increase of GLS at peak exercise was explained by a lower baseline and peak HR, which could not be observed in our study. Neither athletes on semi-recumbent ergometer and treadmill showed significant correlations with HR or differences of mean GLS before and after CPET. Mean GLS was slightly lower after both, semi-recumbent ergometer, and treadmill testing, but did not reach statistical significance. This finding can be explained by higher blood pressure after CPET and consequently higher afterload conditions which was already described in an experimental pig model of aortic banding [36]. It needs to be considered that athletes of different sports are exposed to different forms of physical exercise, and these in turn also have different effects on the cardiovascular system especially on LV remodeling. However, the results of our study are consistent with those of Santoro et al. where GLS was assessed in 27 male water polo players and did not differ significantly before and after 6 repetitions of 100-m freestyle swimming sets [41].

Whereas Gianturco et al. demonstrated a very strong correlation between VO_2max_ and GLS in a cohort of 20 soccer referees and proposed GLS as a specific parameter to assess football referees performance [42], there was no significant correlation between VO_2max_ and GLS in our study. The lack of correlation between GLS and VO2_max_ does not allow conclusions to be drawn about cardiopulmonary exercise capacity based on GLS values in male handball and football players.

The discrepancy between the results of Gianturco et al. and our study is not fully comprehensible. A possible explanation could be the different training conditions between the football referees and the competitive athletes in this study. In addition the measurements of LV deformation were performed by a different vendor compared to this study. Ünlü et al. previously showed that different vendors have a significant impact on tracking feasibility [43].

### Impact of maximum exercise on early post exercise global myocardial work index

Global myocardial work index has proven to be a reliable method for assessing LV function and enables to detect subtle myocardial changes. In noninvasive estimation of LV pressure, GWI based on the pressure strain loop incorporates the current afterload condition and is able to assess LV mechanical function and the myocardial oxygen consumption [44].

Sengupta et al. assessed GWI in 24 recreational athletes before as well as up to a maximum of 2 and 72 h after completing a marathon and found either a decrease in GWI or no change in GWI [45]. A decrease of GWI was attributed to differences in HR and lower LV filling volumes. According to the results of the present study, both aspects could also be observed in professional athletes immediately after CPET, because TTE was performed 5 min after CPET in the present study. GWI has proved to be afterload-independent permitting a more comprehensive assessment of LV systolic function [12], which is beneficial in athletes exposed to different physical exercise. Although systolic blood pressure as a surrogate parameter for afterload conditions was increased in all athletes after CPET. In this study, irrespective of the CPET method, mean GWI did not differ before and after CPET and an individual increase or decrease in GWI was observed in each athlete. This finding lead to the assumption that LV deformation is significantly affected by maximum exercise, whereas the method of exercise testing, semi-recumbent ergometer, or treadmill, does not make a difference.

### Correlation between VO_2max_ and GWI

Tokodi et al. described a moderate correlation between CPET-derived relative VO_2max_ and GWI at rest in a cohort of 20 elite swimmers [46], which was not observed in our study. However, there was a significant correlation between relative VO_2max_ and ΔGWI in both cohorts. This observation can be explained by the fact that well-trained athletes with higher fitness levels show a pronounced increase in GWI after CPET according to their relative VO_2max_, whereas a decrease in GWI after CPET seems to be associated with a lower cardiopulmonary exercise capacity. An explanation for this correlation could be, that athletes with a higher VO_2max_ presumably express higher myocardial load to facilitate the larger oxygen uptake. It can be assumed that **Δ**GWI can be considered as a surrogate parameter to assess the current training condition of athletes.

## Limitations

The number of subjects was limited by the size of the teams in the German handball and football Bundesliga studied at Leipzig University Hospital. However, these highly selected young and healthy competitive athletes highlight the exceptionality of the present cohort. Therefore, the results are not directly applicable to patients with cardiovascular disease. Both semi-recumbent ergometer and treadmill testing could not be performed in all athletes due to their limited time schedule for testing. For the most standardized image acquisition possible, TTE was performed in handball and football players 5 min after CPET and not at maximum exercise. In addition, the determination of LV deformation parameters by 2D speckle tracking analyses is often erroneous at very high heart rates, so that most likely some LV segments would not have been tracked reliably. The modality of incremental cardiopulmonary exercise test was predetermined by the respective medical team leader.

## Conclusion

VO2_max_ is considered an important indicator of athlete’s training condition and maximum performance capacity. In the present study we confirmed that maximum exercise has a significant effect on LV deformation, irrespective of the exercise method.

Further, we were able to demonstrate a significant correlation between ΔGWI and VO2_max_ directly after CPET, so that the current training condition or maximum performance capacity of an athlete might also be estimated by a single modern imaging parameter instead of only VO2_max_. Further studies are needed to clarify whether athletes who demonstrate a decrease in GWI and a lower VO2_max_ after CPET have a higher performance potential and thus their maximal performance capacity can be further improved. If so, GWI could be used as a modern imaging parameter to characterize the athletes’ maximum performance capacity, which would considerably enrich and simplify individual performance diagnostics.

## Data Availability

The authors confirm that the data supporting the findings of this study are available within the article.
